# Study of Association of Dyslipidemia in Male Androgenetic Alopecia Patients in a Tertiary Care Hospital

**DOI:** 10.7759/cureus.51741

**Published:** 2024-01-06

**Authors:** Harshatha Sathyanarayanan, Murugan Sundaram

**Affiliations:** 1 Dermatology, Sri Ramachandra Institute of Higher Education and Research, Chennai, IND

**Keywords:** metabolic syndrome, cardiovascular disease, lipid profile, androgenetic alopecia, dyslipidemia

## Abstract

Introduction: Androgenetic alopecia (AGA) is a hereditary and androgen-dependent progressive thinning of the scalp hair in a defined pattern. Several studies have reported an association between dyslipidemia and AGA. However, scarce data is available on association between severity of AGA and dyslipidemia. Hence, we performed a study to assess the clinical, epidemiological profile in male AGA patients and to compare lipid parameters among AGA cases and non-AGA controls to evaluate dyslipidemia association.

Materials and methods: This is a prospective case-control study included 108 age-matched AGA cases and non-AGA controls between 19-40 years. AGA was clinically diagnosed, and grading was done according to Norwood-Hamilton Classification. Fasting Lipid parameters for both cases and controls were determined using standard laboratory methods.

Results: Among the cases and controls, the mean age was 26.20±5.353 years. There was a statistically significant association between AGA and mean total cholesterol (TC) (P<0.001), mean high-density lipoprotein (HDL) (P<0.001), mean low-density lipoprotein (LDL) (P<0.001) and mean cholesterol/HDL ratio (P<0.001), except for mean triglycerides (TG) (P=0.443). Grade 4 was the commonest grading (20.4%). As the severity of AGA increased, the lipid parameters were significantly deranged. It was evident Grade 4 onwards with statistically significant derangement in TC (P<0.001), TG (P=0.005), HDL (P=0.002), LDL (P <0.001) and cholesterol/HDL(P<0.001).

Conclusions: AGA was found to be significantly associated with dyslipidemia and more common among severe grades. AGA could be a cutaneous marker of underlying systemic illness. Early screening for dyslipidemia is beneficial in patients with AGA.

## Introduction

Male pattern baldness, also known as androgenetic alopecia (AGA) is the most common cause of hair loss in men. It describes a form of hair loss that occurs in a generally distinctive pattern and is characterized by a progressive decline in hair-fibre production by hair follicles and their eventual miniaturization, finally leading to baldness [1]. AGA commonly begins by 20 years of age and affects nearly 50% of men by the age of 50 and affects 80% of men by the age of 70 [2].

AGA predominantly affects men and occasionally women, with significant negative impact on their social and psychological well-being and more likely to cause emotional distress. It has been proven to be associated with many comorbidities like myocardial infarction, metabolic syndromes, dyslipidemia and hypertension [3-13]. Dyslipidemia is a proven predisposing factor to coronary artery disease (CAD). Hence AGA may represent an evolving underlying dyslipidemia with future cardiovascular risk like CAD.

The scope of the study and generation of data will be significant in assessing the morbidity and counselling the patients in order to screen for underlying comorbities. Due to scarcity of studies in establishing an association between early onset AGA and dyslipidemia, the study was carried out in our dermatology department in order to evaluate clinico-epidemiological profile of AGA and to determine if AGA could be a cutaneous marker/precursor of underlying dyslipidemia. In the present study, we have also evaluated the association between the grading pattern of AGA and the lipid parameters.

## Materials and methods

Study design

This is a case-control type of study conducted at Sri Ramachandra Institute of Higher Education and Research, Chennai, over a period of two years from September 2017 to September 2019 with ethical committee clearance obtained from from the aforementioned institution (Ref- CSP-MED/17/AUG/37/97).

Study population 

The study group included male AGA and normal (non-AGA) subjects in the age group of 19-40 years with a sample size of 100 cases and 100 controls, respectively. Subjects fulfilling the inclusion criteria on enrollment, who have consented for the study.

Data collection

After obtaining informed consent from all the subjects, detailed case history will be taken. Demographic data including age, gender, history of any pre-existing comorbidities were taken for cases and controls. Each enrolled patient was subjected to a thorough clinical, dermatological and systemic examination. AGA was diagnosed based on clinical examination. Grading of AGA will be done for the cases according to Norwood-Hamilton Classification (1975), a standard classification scheme. Blood samples were drawn for evaluation of lipid parameters for both cases and controls following 12 hours fasting status and serum levels of triglycerides (TG), total cholesterol (TC), high-density lipoprotein (HDL), and low-density lipoprotein (LDL) were determined using standard laboratory methods. BMI and anthropometric measurements were determined for all the patients.

Data analysis

Data was analysed using SPSS software version 16.0. Descriptive statistics like mean ± standard deviation was calculated for quantitative variables and qualitative variables. Categorical data were represented in terms of frequency and percentage. Mean values were compared across the groups by means of t-test and proportions were evaluated using Pearson’s Chi-square test. One-way ANOVA analysis was utilised to evaluate the significance for more than two groups. The differences were considered statistically significant if p-value was < 0.05.

Inclusion and exclusion criteria

Inclusion criteria for the study included male AGA subjects aged from 18 to 40 years. 100 age-matched male individuals with normal hair status without AGA, having other minor skin problems without attributable hair loss in the age group of 18 to 40 years, who have consented for the study were included as controls.

Cases associated with other conditions like seborrheic dermatitis, psoriasis, telogen effluvium, anagen effluvium, alopecia areata, chemotherapy/other drugs, due to trauma, on dyslipidemia/hormone replacement therapy with testosterone, corticosteroid therapy, hypothyroidism were excluded. Patients ≤ 18 and ≥ 40 years of age and not willing to participate in the study were excluded.

## Results

A total of 108 AGA cases and age-matched non-AGA controls were included in the study. In the following table, we have compared various parameters between cases and controls (Table 1).

**Table 1:** Comparison between cases and controls AGA: Androgenetic alopecia; TC: Total cholesterol; TG: Triglycerides; HDL: High-density lipoprotein; LDL: Low-density lipoprotein

Parameters	Cases	Controls	P value
No. of cases (n)	108	108	NA
Mean age	26.20 ± 5.353	26.09 ± 5.363	0.879
Duration of hair loss	4. 3 years	NA	NA
Age at onset	21.79 ± 3.796	NA	NA
Family history of AGA (n(%))	95 (88.8%)	12 (11.1%)	< 0.001
Alcohol consumption (n(%))	40 (37%)	32 (29.6%)	0.248
Smokers (n(%))	29 ( 26.9%)	18 (16.7%)	0.070
BMI (kg/m^2^)	23.299 ± 3.099	23.852 ± 3.386	0.216
Blood Tests ( mean ± SD)			
TC (mg/dl)	205.06 ± 48.407	182.56 ± 51.723	< 0.001
TG (mg/dl)	132.88 ± 45.059	121.81 ± 14.787	0.443
HDL (mg/dl)	38.46 ± 7.097	45.81 ± 11.272	< 0.001
LDL (mg/dl)	129.90 ± 29.346	110.94 ± 35.697	< 0.001
Cholestrol/HDL	5.5491 ± 2.053	4.0256 ± 1.991	< 0.001

In this study, subjects with AGA were categorized based on Norwood-Hamilton classification. The most common grading of alopecia was Grade 4 with 20.4% cases followed by Grade 4A (37.4%) and 5 (13.8%) (Table 2).

**Table 2:** Grading of AGA based on Norwood-Hamilton classification AGA: Androgenetic alopecia

Grading	1	2	2A	3	3A	3V
No. of cases	7 (6.5%)	7 (6.5 %)	2 (1.9 %)	6 (5.6%)	5 (4.6%)	4 (3.7%)
Grading	4	4A	5	5A	6	7
No. of cases	22 (20.4%)	18 (16.7%)	15 (13.8%)	8 (7.4%)	6 (5.6%)	8 (7.4%)

On further categorisation of the subjects into mild, moderate and severe types of AGA, which are Grades 1, 2; Grades 2A, 3, 3A, 4; and Grades 4A, 5, 5A, 6, 7, respectively, severe AGA was found to be the commonest presentation (50.9%) than mild (13%) and moderate types (36.2%) [14].

On comparing the lipid profile parameters with grading of AGA, we observed that as the severity of AGA progressed, the lipid levels were found to be significantly deranged (Table 3). The lipid parameters were compared among the mild, moderate and severe grades of AGA and found that the mean TC, mean LDL, mean cholestrol/HDL was significantly higher and mean HDL values were significantly lower with P value <0.05. Mean TG values were found to be normal (P=0.005).

**Table 3:** Summary of comparison of parameters based on severity of AGA AGA: Androgenetic alopecia; TC: Total cholesterol; TG: Triglycerides; HDL: High-density lipoprotein; LDL: Low-density lipoprotein

Variables	Mild	Moderate	Severe	P value
BMI	22.0994 ± 2.925	23.2498 ± 3.007	23.8930 ± 3.226	0.152
TC	169. 44 ± 46.54	201. 47 ± 51.261	225.81 ± 33.082	< 0.001
TG	102.69 ± 25.705	133.15 ± 46.836	145.54 ± 43.574	0.005
HDL	42.06 ± 3.855	39.49 ± 7.630	35.38 ± 6.247	0.002
LDL	107. 81 ± 26.202	128. 02 ± 33.594	142.24 ± 14.210	< 0.001
Cholestrol/HDL	4.0250 ± 1.158	5.2364 ± 1.716	6.673 ± 2.251	< 0.001

## Discussion

AGA is a genetically determined disorder due to elevated levels of androgens and increased AR binding in genetically predisposed men. It has been proven to be associated with many comorbidities like myocardial infarction, metabolic syndromes, dyslipidemia and hypertension. Hence, AGA may represent an evolving underlying dyslipidemia with future cardiovascular risk like CAD and other complications [15]. In the present study we have assessed the serum lipid profile in order to explicate the association of dyslipidemia in subjects with AGA. 

The mean age of patients with AGA in the present study was 26.20±5.353 years which was similar to studies conducted by Chakrabarty et al. and Banger et al. who had 26.44±2.64 and 27.03±5.36, respectively [16,17]. The mean age of onset in this study was slightly lower than the previous studies by Bakry et al. and Nargis et al. which showed 24.74± 4.24 and 23.0 years, respectively [12,18]. In this study, the paternal history (72.2%) and maternal history (15.7%) was slightly higher than Nargis et al. and almost similar to the Shegal et al. study. However only 12% of patients in this study did not have family history which was similar to Shegal et al. (15%) and in contrast to Nargis et al. (35.2%). This showed that patients who had both paternal and maternal history of AGA are more likely to develop the same. Especially, the prevalence of paternal alopecia has more impact on AGA expression than maternal alopecia [12,19]. Various genetic and environmental factors are implicated in the pathogenesis of AGA. Among which AR gene, an X-linked recessive gene has been cited as the prime prerequisite in the genetic sensitivity of the primary and secondary hair follicles to DHT that lead to hair follicular miniaturization [20]. In this study, there was no significant association between AGA and alcohol consumption (37%) and smoking history (26.9%). These findings were similar to the Severi et al. study and in contrast to that by Park et al. and Nargis et al., who showed a statistically significant association between AGA and alcohol consumption, AGA and smoking. It has been hypothesized that smoking increases the testosterone levels thereby increasing the risk of AGA progression [9,12,21]. However, our study did not substantiate the same. In the present study, there was no statistically significant association (P=0.216) with BMI on comparison with controls, which was also similar to the Arias-Santiago et al. study, but in contrast to the Park et al., Bakry et al. and Gopinath et al. studies that have shown a significant association between AGA and BMI [7,18,22]. This may be attributed to the inclusion of relatively younger age group of patients in our study population. The frequency of obesity is clearly related to age and patients whose age range between 25 and 34 years will have second lowest percentage of obesity in adult age group [23,24].

In the present study, the mean lipid values were compared between AGA cases and controls and it showed statistically significant association between AGA and mean TC, LDL, HDL, cholestrol/HDL ratio but it was statistically insignificant between AGA and mean serum TG. These findings were in contrast with Chakrabarty et al., Banger et al. and Park et al., who showed statistically significant association between AGA and the lipid parameters [9,16,17]. Guzzo et al. showed statistically insignificant association between AGA and lipid levels [10]. Acibucu et al. showed significant association between TC, TG and AGA but serum LDL and HDL levels did not show any significant association between AGA cases and controls [25]. The inconsistency in TGs when compared to various studies can be elucidated by the fact that the lipid parameters are associated with diverse factors that includes age, sex, genetics, environment and majority of patients in this study belong to an age group of less than 40 years. 

Association of AGA and cardiovascular disease could probably be explained the fact that 5 α-reductase enzyme is present in walls of blood vessels and the heart and the DHT receptors are engaged in the vascular smooth muscle proliferation which represents a basic feature of atherosclerosis with lipid deposition [26]. And also androgen hormones have been proven to reduce the HDL-C levels in experimental studies [27]. Higher levels of TG and lower levels of HDL-C were found to be associated with the conversion from atheroma to atherothrombosis [17]. Our study substantiates the same, we have proven a statistically significant association between AGA and lipid parameters. In the present study, Grade 4 (20.4%) was the commonest presentation that was in contrast to Gopinath et al., which showed Grade 3 (18.8%) as the commonest grading, and Salman et al. with Grade 3 vertex (28.9%) as the most common presentation. 

Dyslipidemia is said to be associated with AGA as reported by various studies [9-11]. However, very few studies have compared the severity of AGA with lipid parameters which is the novelty of the present study, as the association of dyslipidemia and AGA were reported earlier. In this study, we have compared the severity of AGA with lipid parameters in order to evaluate if there is an association. In the present study, we found a statistically significant association between the severity of AGA and the lipid parameters. As the severity of AGA progresses there was a significant derangement in TC, HDL, LDL, cholestrol/HDL and insignificant association with TG. It is in contrast to Park et al., who showed a statistically significant association with TC, HDL and insignificant association in TG and LDL levels (Table 4). This discrepancy between the studies could be because the Park et al. included patients with AGA who were on medication for dyslipidemia [9]. As the severity of AGA increases there is derangement of lipid parameters, which may be attributed to the higher level of circulating androgen hormones in patients with AGA, but the exact pathogenesis has to be further studied [9]. 

**Table 4:** Comparison of lipid profile parameters based on severity of AGA with previous studies AGA: Androgenetic alopecia; TC: Total cholesterol; TG: Triglycerides; HDL: High-density lipoprotein; LDL: Low-density lipoprotein

Lipid parameters	Severity of AGA	Present study	Park et al. [9]
TC	Mild	169.44 ± 46.54	193.63 ± 40.52
	Moderate	201.47 ± 51.261	188.43 ± 37.89
	Severe	225.81 ± 33.082	181.35 ± 39.28
TG	Mild	102.69 ± 25.705	123.68 ± 70.44
	Moderate	133.15 ± 46.836	126.49 ± 70.80
	Severe	145.54 ± 43.574	151.03 ± 210.01
HDL	Mild	42.06 ± 3.855	57.69 ± 15.11
	Moderate	39.49 ± 7.630	56.18 ± 16.39
	Severe	35.38 ± 6.247	53.75 ± 114.78
LDL	Mild	107.81 ± 26.202	107.04 ± 34.49
	Moderate	128.02 ± 33.594	105.45 ± 32.29
	Severe	142.24 ± 14.210	106.57 ± 35.02
Chol/ HDL	Mild	4.0250 ± 1.158	-
	Moderate	5.2364 ± 1.716	-
	Severe	6.673 ± 2.251	-

The limitation of our study was small sample size. A larger sample size maybe needed to establish a significant relationship between grades of AGA and dyslipidemia. Insulin resistance, metabolic syndrome, and diabetes were not evaluated in the present study where there exists a proven association with AGA from various studies. 

## Conclusions

It has been widely accepted that dyslipidemia is a metabolic disorder of elderly age group. But recently several studies have proven an association between younger men with AGA and dyslipidemia. Our present study showed that AGA in younger men is associated with dyslipidemia. Therefore, the health professionals need to emphasize on the grading of alopecia and to create an awareness among the younger population with AGA regarding the risk of developing dyslipidemia and its consequences in the future that includes cardiovascular diseases, metabolic syndrome, diabetes and hypertension. Therefore, treating the AGA patients may not be under the sole purview of dermatologist alone but a multidisciplinary initiative involving physician and nutritional expert opinion as well. Patient counselling to draw attention to these correctible parameters and comorbidities is essential part of ensuring a good quality of life in AGA. Significant lifestyle modification and dietary rebalancing are an essential part of the holistic management protocol in therapy for AGA.
